# Gallbladder Perforation Due to *Ascaris lumbricoides* in a Pregnant Woman and 6 Year Old Girl from Afghanistan: Case Report

**Published:** 2019

**Authors:** Sayed Hussain MOSAWI, Abdolhossein DALIMI, Mohammad Ali CHARKHI, Omer BAARAE, Amanullah DARMAN, Moqadas MOSAVI, Mohammad WALI BARYAL, Hela STANIKZAI

**Affiliations:** 1. Department of Parasitology, Faculty of Medical Sciences, Tarbiat Modares University, Tehran, Iran; 2. Medical Sciences Research Center, Ghalib University, Kabul, Afghanistan; 3. Department of Pediatric Surgery, Indira Gandhi Institute for Child Health, Kabul, Afghanistan; 4. Department of Surgery, Isteqlal Hospital for Adult Health, Kabul, Afghanistan; 5. Faculty of Medicine, Khatam Al-Nabieen University, Kabul, Afghanistan

**Keywords:** *Ascaris lumbricoides*, Gallbladder, Pregnancy, Afghanistan

## Abstract

Ascariasis is an important and prevalent geo-helminth infection, especially in underdeveloped countries such as Afghanistan, where public health problems and devastation of hygienic infrastructures usually lead to the higher incidence of the infection. Here, we report two cases of intestinal obstruction that accompanied with biliary ascariasis due to *Ascaris lumbercoides* in pregnant women and a 6-year-old girl from Afghanistan. Biliary ascariasis can be associated with cystic duct obstruction, gallbladder dilation, acute cholecystitis, acute cholangitis, obstructive jaundice, and biliary colic.

## Introduction

Ascariasis is one of the most common parasitic infections, which approximately infects 25% of the global population with one million cases annually (1). Conducive climate and poor sanitary conditions in subtropical and tropical regions entail the endemicity of the infection (2). This geohelminth has a direct life cycle with adults in the intestinal lumen. High parasite burden renders worm aggregation in the intestine and the subsequent obstruction, volvulus or intussusception of the foci; also, the parasites may migrate into the bile ducts and even perforate the gallbladder (1, 3).

Under-developed countries such as Afghanistan always suffer from soil-transmitted helminthiases, particularly due to weak socioeconomic status, low hygienic and sanitary conditions as well as insufficient public awareness (4, 5). Hence, high prevalence of parasitic infections like ascariasis is anticipated.

Here, we documented two case reports of intestinal and biliary *Ascaris* infection in pregnant women and a 6-year-old girl from Afghanistan.

## Case Reports

### Ethical consideration

Approval of ethics application was taken from Medical Ethic Committee of Ghalib University, Kabul, Afghanistan. All the protocols used in this study were in accordance with the approved guidelines and informed agreement was taken from the patients.

### Case 1

A 17-year-old woman who was pregnant with acute abdominal pain came to the Outpatient Department of Esteqlal Hospital, (Kabul City, Afghanistan) in 2017. She was from Bamyan Province in the center of Afghanistan. She was suffering since eight days ago intensified last two days and was mostly localized at right upper quadrant of the abdomen. She also complained of vomiting, nausea and anorexia for one day.

Based on physical examination, the vital signs were recorded as follows: blood pressure 100/60 mmHg, respiratory rate 22 per minute, body temperature 37 °C and pulse rate of 80 beats per minute. Moreover, the patient had 22 wk pregnancy. Abdomen was shown to be normal in the inspection. The bowel sounds were present in auscultation. Tender and painful upper right quadrant were discovered during palpation. Furthermore, dullness was revealed in abdominal percussion. Other body systems were normal at the examination.

Accordingly, the clinician claimed cholecystitis as provisional diagnosis. Ultrasonography showed that there was a long linear-shaped structure in the lumen of common bile duct (CBD), resembling a round worm. Laboratory examination indicated that hemoglobin was 12 gr/100 ml, white blood cells, TLC (total leukocyte) 12000, many epithelial cells in urine, 12–15 white blood cells in urine, 0.94 mg/dl total bilirubin, 0.21 mg/dl direct and 0.78 mg/dl indirect bilirubin, 26 U/L alanine aminotransferase (ALAT), 19 U/L aspartate transaminase (AST) and 179 U/L alkaline phosphatase.

By taking into consideration of sterile condition of OT (Operation Theater) and under spinal anesthesia in supine position, after preparation and draping, the right upper para medium was opened, and the gallbladder was received that two *Ascaris* worms were available.

**Fig. 1:**
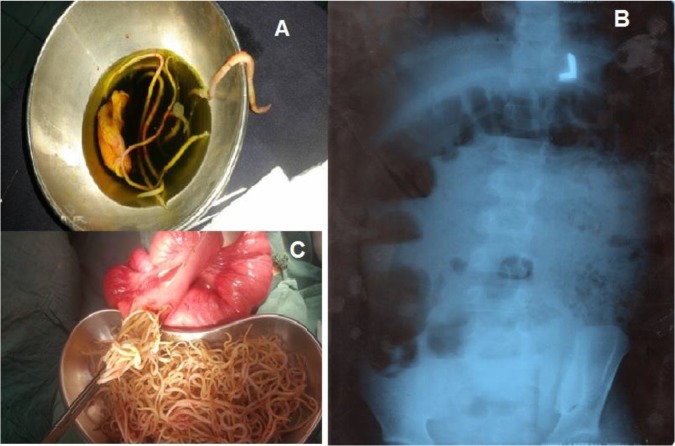
**A:** Removed gallbladder with detected worms (pregnant woman), **B:** Intestinal soap bubble sign and multiple air fluid level (Right) and railway appearance (Left) and **C:** Removed worms from the gut lumen (the 6 yr old girl)

Despite the CBD not dilated, foreign body was touchable. CBD exploration was done on the patient, and some *Ascaris* worms were removed (Fig. 1, A). After several lavage, a T-tube was placed and stabilized. Before implementation of CBD explores, cholecystectomy was operated on the patient, and the gallbladder was sutured by two chromic sutures. Moreover, one drain was placed in the gallbladder (lavage). Eventually, the skin layers were restored step by step and patient was transferred to the ICU ward after anesthesia and performing surgery.

### Case 2

A six years old female patient from Barakebarak district, Lugar Province, west of Afghanistan, was admitted to the Emergency Department of the Indira Gandhi Hospital in Kabul, Afghanistan, on 12 Jul 2017, with acute abdominal pain, vomiting, and abdominal expansion for 3 d. On physical examination, she was well-nourished. Her oral temperature was 37.5 °C, blood pressure was 100/60 mmHg, and the pulse was regular with a rate of 120 beats per minute. Respiratory rate was 35 per minute and there observed bilateral air entry with no added sounds but slight respiratory efforts. Cardiovascular examination demonstrated normal S1 and S2 with no murmur, and central nervous system evaluation revealed no neurological deficits. Abdominal examination revealed tenderness and rigidity in the central and mid-abdomen. Moreover, the patient had edema, vomiting, and abdominal expansion. Auscultation revealed a silent abdomen or minimal peristalsis.

At the time of admission, laboratory investigations were done. The red blood cell count was (3.4–3.8×10/μl) and hemoglobin level was 11.5gr/100ml, total leukocyte count: 13400, and bleeding time (2′ 10″), also clotting time (4′ 20″). The consequence tests of HBs & HCV were negative. Other blood tests were negative for acquired immune deficiency syndrome (AIDS), viral hepatitis and metabolic panel (M.P.) Moreover, her blood group was A/Rh^+^ and about 200 cc blood was transferred to the patient.

Abdominal ultrasound showed that, the gallbladder was normal in size and shape. Multiple alive *Ascaris* worms were seen within the gallbladder lumen. No lithiasis or focal mass was present. Common duct was normal, being 3 mm in diameter, whereas the normal diameter is 6 mm. Liver, kidney, spleen, and pancreas were macroscopically normal. There was not observed any para aortic lymphadenopathy as well as no free fluid in the peritoneal cavity. Furthermore, X-ray revealed the intestinal obstruction with two signs, including 1) soap bubble sign and multiple air-fluid levels and 2) railway appearance (Fig. 1, B).

All these findings indicated to intestinal ascariasis. In the following, nasogastric tube, IV infusion and antibiotic therapy (Cefotaxime 500m g IV) once 30–90 min before starting the procedure and the Metronidazole 500mg/100mL infusion was performed for the patient. After that, the patient moderately became ready for operation, general anesthesia was induced and the abdomen was opened by midline short incision. *Ascaris* worms were mostly concentrated at the median part of the ileum, caused intestinal obstruction (Fig. 1, B). Enterotomy and milking method was used to remove worms from the lumen. After dislodgment of 3.5 kg of worms from the intestine (Fig. 1, C), the lumen was stitched up and the patient went to recovery with normal vital signs. Additionally, biliary ascariasis was treated with albendazole. After 3 d the patient acquired the fluid intake. Seven days post-operation, the girl was discharged from the hospital in a good condition.

## Discussion

In the ascariasis abdominal pain, anorexia, nausea, vomiting, fever, dehydration and abdominal distension are the most common symptoms of the disease. Moreover, intestinal obstruction may happen due to the high burden of parasite in children. The related manifestations are pneumonia and intestinal, appendicular, hepatobiliary and pancreatic ascariasis. The confliction of gallbladder, is particularly due to wandering worms within the body in case of high parasite load in the intestine and it is also related to worm size, sex and age of the host (6–8). In pregnancy, the risk of biliary ascariasis increases because of the high level of progesterone and estrogen hormones that influence Oddi sphincter, as previously proved in animal models. These hormones facilitate the migration of worms into biliary ducts. Furthermore the mentioned hormones lead to poor draining of the gallbladder through the second and third trimesters of pregnancy (9).

The patient in this study was also in her second trimesters of pregnancy (22 wk). Laboratory tests, CT scan, MRI, MRCP (magnetic resonance cholangiopancreatography), ERCP (endoscopic Retrograde Cholangiopancreatogram) and abdominal ultrasound are beneficial for diagnosis. The ultrasound is available, sensitive, specific, safe and noninvasive for the diagnosis of biliary ascariasis in pregnant women. It can represent the worms as a long, linear or curved with no shadowing echogenic strip and reveals a characteristic movement of worms in the biliary tree. Similar finding was seen in the case of present study. Treatment of the biliary ascariasis is mainly by endoscopy or surgery instead of using anthelminthic drugs because of the low excretion of this in bile (less than 1%) (10–12). The usage of pyrantel pamoate and piperazine citrate are harmless in the late pregnancy, while mebendazole or albendazole are contraindicated. Biliary ascariasis can result in severe problems for the mother and fetus. All the associated surgical procedures may increase the risk of fetal loss and these procedures should be taken after delivery. According to reports, maternal mortality rates in pregnancy have been about 21% due to ascariasis (9). In the treatment procedure belonging to first case, removal of worms and cholecystectomy were used.

In case of bowel obstruction, dilated and air-filled intestinal loops are observed along with several air-fluid levels, indicating the railway track sign (13). Similar findings were seen in our second case. In case of jejunal obstruction with multiple masses, enterotomy and worm removal is the method of choice. Whenever the intestinal wall is thin, for instance, in volvulus cases, milking is avoided due to serosal tears and enterotomy would be better. Herein, in the second case we resected the involved section of intestine and anastomose the rest (14, 15).

Today, the control of ascariasis is not as difficult as before, since the availability of anthelmintic drugs. Public health can play a significant role in disrupting the transmission of the parasite cycle by mass chemotherapy, promotion of health education and the improvement of sanitation in Afghanistan. The main key to eliminating ascariasis in this country is the elevation of sewage system and the other organizations related to disposal of human feces.
